# Rab31 promotes the invasion and metastasis of cervical cancer cells by inhibiting MAPK6 degradation

**DOI:** 10.7150/ijbs.63388

**Published:** 2022-01-01

**Authors:** Yujie Huang, Ruijuan Liu, Xuechao Han, Xiaoyan Hou, Yonghao Tian, Weifang Zhang

**Affiliations:** 1 Department of Microbiology, School of Basic Medical Sciences, Cheeloo College of Medicine, Shandong University, Jinan, Shandong, China.; 2 Department of Orthopedics, Qilu Hospital of Shandong University, Jinan, Shandong, China.

**Keywords:** Cervical cancer, HPV, Rab31, MAPK6, Metastasis

## Abstract

Persistent infection with high-risk human papillomavirus (HPV) is the main risk factor for cervical cancer. Our mass spectrometry data showed that the Ras-associated binding protein Rab31 was upregulated by HPV; however, little is known regarding the role of Rab31 in the metastasis of cervical cancer cells. In this study, we showed that Rab31 was highly expressed in cervical cancer tissues and cells, and both HPV E6 and E7 promoted the expression of Rab31. Rab31 knockdown inhibited while Rab31 overexpression promoted the migration and invasion capabilities of cervical cancer cells. Additionally, Rab31 knockdown inhibited the epithelial-mesenchymal transition (EMT) and cytoskeletal rearrangement in cervical cancer cells. Furthermore, Rab31 interacted with mitogen-activated protein kinase 6 (MAPK6), and Rab31 knockdown inhibited the expression of MAPK6, which was mainly localized in the cytoplasm. More importantly, Rab31 knockdown promoted and Rab31 overexpression inhibited MAPK6 degradation. Accordingly, MAPK6 overexpression restored the decreased migration potential caused by Rab31 knockdown. Finally, a xenograft mouse model showed that Rab31 knockdown in cervical cancer cells led to reduced tumor growth and impaired lung and liver metastasis *in vivo*. In conclusion, Rab31 plays a crucial role in cervical cancer metastasis by inhibiting MAPK6 degradation. Thus, Rab31 may serve as a novel therapeutic target to manage cervical cancer.

## Introduction

Cervical cancer is the fourth most common malignancy in women worldwide. Globally, approximately 604,000 new cases and 342,000 deaths of cervical cancer occurred in 2020. Nearly all cervical cancers are caused by persistent human papillomavirus (HPV) infection. HPV is a non-enveloped double-stranded DNA virus that mainly infects the human epidermis and mucosal squamous epithelium 1. HPV16 and 18 type infections account for 70% of cervical cancer and precancerous cervical lesions 2. The early oncoproteins E6 and E7 contribute to cancer progression 3. The E7 protein binds to and degrades Rb protein family members 4, resulting in the release and activation of the E2F1 transcription factor 5. The E6 protein promotes ubiquitin-dependent degradation of the tumor suppressor p53 6. However, many unknown cellular targets of HPV E6 and E7 proteins must be identified.

Many malignant cancers are accompanied by invasion and metastasis of cancer cells, which is a major cause of cancer-related death. Cancer cells enter the bloodstream or lymph system, spread throughout the body and establish secondary tumors in distant organs 7. Local infiltration cervical cancer often involves down the vaginal wall. After local invasion of cancer cells, they invade the lymphatic vessels and circulate through this system. Blood metastasis is rare and advanced cancers can transfer to the lungs, liver or bones 8. Cervical cancer invasion and metastasis are highly complex biological processes. Studies have shown that HPV16 E6 downregulates Na^+^/H^+^ exchanger regulatory factor 1 (NHERF1), thereby increasing actin cytoskeleton protein ACTN4 levels to enhance actin polymerization and promote cervical cancer cell migration and invasion 9. Zhang HR *et al* showed that HPV16 E6/E7 enhanced myosin 1b expression by upregulating c-MYC, thereby increasing the activity of matrix metalloproteinase 1/9 (MMP1/MMP9), degrading the extracellular matrix (ECM), and increasing CaSki cell invasion and transfer 10. The prognosis of cervical cancer is far from satisfactory, and the 5-year survival of patients with stage IV cervical cancer classified by FIGO is only 5-15% 11. Thus, exploring the molecular mechanisms of the invasion and metastasis of cervical cancer is essential to understand the progression of cervical cancer and development of novel effective treatments.

Rab31 belongs to the Rab family, which is the largest Ras superfamily of small molecule GTP binding proteins 12. Rab31 is mainly localized in the trans-Golgi network and endosomes, regulating material transport between the Golgi/trans-Golgi network and plasma membrane/endosome 13, such as the type 4 glucose transporter (GLUT4) 14 and epidermal growth factor receptor (EGFR) 15. Accumulating evidence has shown that Rab31 is widely involved in carcinogenesis and is related to the prognosis of various cancers. High expression of Rab31 is frequently observed in breast cancer tissues 16 and is associated with enhanced cell proliferation 17. Rab31 expression is increased in glioblastoma multiforme 18. Elevated Rab31 in pancreatic cancer is associated with reduced overall survival 19. Rab31 is related with the malignant behavior of gastric cancer and osteosarcoma 20, 21. Recently, our mass spectrometry results show that Rab31 expression is upregulated in HPV16 E6- or E7-expressing cells (data not shown). However, little is known regarding the tumor biological effects of Rab31 in cervical cancer.

In the present study, we examined the Rab31 expression levels in cervical cancer tissues and cells. Next, cervical cancer cells with Rab31 knockdown or overexpression were constructed to explore the role of Rab31 in cervical cancer metastasis and underlying molecular mechanism. We identified mitogen-activated protein kinase 6 (MAPK6) as a binding partner of Rab31 and demonstrated that Rab31 inhibited MAPK6 degradation. Additionally, we observed significantly impaired tumor growth and metastatic dissemination of Rab31-knockdown cervical cancer cells to the lung and liver in a xenograft mouse model. Our data provide novel insights into the role of Rab31 in cervical cancer progression.

## Materials and Methods

### Patient tissue samples and cell culture

Fifty-five non-cancer cervical tissue samples and 66 cervical cancer tissue samples were collected from Qilu Hospital of Shandong University and used for RNA extraction, reverse transcription and real-time quantitative PCR. Non-cancer cervical tissues were obtained from patients with chronic cervicitis or uterine fibroids undergoing total hysterectomy. This study was approved by the Ethics Committee of Shandong University School of Medicine.

The human immortalized keratinocytes cell line HaCaT, and the cervical cancer cell lines HeLa, SiHa, CaSki and C33A were cultured in Dulbecco's modified Eagle medium (DMEM) supplemented with 10% fetal bovine serum (FBS). Human telomerase reverse transcriptase-expressing human retinal pigment epithelial cells (RPE1) were maintained in Ham's F12-DMEM medium (1:1) plus 10% FBS. All the cell lines were purchased from ATCC and cultured in a humidified incubator with 5% CO_2_.

### siRNA and plasmid transfection

Specific small interfering RNAs (siRNAs) targeting Rab31 (si-Rab31), MAPK6 (si-MAPK6) and negative control siRNA (si-NC) were purchased from GenePharma. Cells were seeded in 6-well plates, and the next day the cells were transfected with 20 nM siRNAs using Lipofectamine 2000 according to the manufacturer's instructions. After 6 h, the medium was removed and replaced with fresh culture medium containing 10% FBS. For rescue experiments, the MAPK6 expression plasmid pCMV3-HA-MAPK6 was transfected into cells 24 h after Rab31 siRNA transfection. For overexpression experiments, C33A cells were transfected with the Rab31 expression plasmid pENTER-C-Flag-His-Rab31.

- si-Rab31: 5'- GGAUCACUUUGACCACCACAACTT -3';
- si-MAPK6: 5'- GGCUUUUCAUGUAUCAGCUTT-3';
- si-NC: 5'- UUCUCCGAACGUGUCACGUTT -3'.

### Lentivirus infection

Cells were seeded in 6-well plates and cultured in medium for 24 h before lentivirus infection. When the cells were 30-40% confluent, lentivirus (expressing green fluorescent protein, GFP) of sh-Rab31-1 (sh-Rab31), sh-Rab31-2, or sh-NC was added to each well of HeLa (MOI=30) and SiHa (MOI=20) cells using 5 μg/mL of polybrene. The cells were transferred to a 10 cm Petri dish, and 1 μg/mL of puromycin was added to the medium to the selected stably transfected HeLa and SiHa cell lines with Rab31 knockdown.

### Quantitative Reverse-Transcription PCR

TRIzol was used to lyse the cells, and chloroform was added to extract the cellular RNA. After measuring the RNA concentration, the reverse transcription kit HiScript^®^ Q Select RT SuperMix for qPCR was used to reverse transcribe the RNA to cDNA. A HiScript II One Step RT-PCR SYBR Green Kit was used to amplify the products and monitored using a CFX96 Touch Real-Time PCR detection system. The PCR primers were as follows:

- Rab31-F: AGGAATACGCTGAATCCATAGG;
- Rab31-R: TTCCTTGAAAGAGCTCTTCGAT;
- MAPK6-F: TAAAGCCATTGACATGTGGG;
- MAPK6-R: TCGTGCACAACAGGGATAGA;
- GAPDH-F: GCACCGTCAAGGCTGAGAAC;
- GAPDH-R: GCCTTCTCCATGGTGGTGAA.

PCR experiments were performed according to the following conditions: initial denaturation at 95 °C for 30 sec, denaturation at 95 °C for 10 sec, extension at 60 °C for 30 sec for 40 cycles.

### Western blotting

Total cellular proteins were extracted using Cell Lysate RIPA lysis buffer with phenylmethanesulfonyl fluoride (PMSF) and protease inhibitor at a 100:1:1 ratio. Proteins were separated by 10% SDS-PAGE and transferred to PVDF membranes. After blocking in 5% skimmed milk, the membranes were incubated with primary antibodies against EMT Antibody Sample Kit (1:1000; 9782; Cell Signaling), Lamin A/C (1:1000; 4777; Cell Signaling), MAPK6 (1:2000; ab53277; Abcam), MMP2 (1:1000; 40994; Cell Signaling), MMP9 (1:1000; 13667; Cell Signaling), p53 (1:2000; 10442-1-AP; Proteintech), pRb (1:1000; 554136; BD Biosciences), Rab31 (1:1000; 16182-1-AP; Proteintech), GAPDH (1:1000; 10494-1-AP; Proteintech), β-tubulin (1:1000; 86298; Cell Signaling), Flag (1:1000; 20543-1-AP; Proteintech) and HA (1:1000; 66006-2-Ig; Proteintech). The goat anti-mouse HRP-conjugated secondary antibody (ZB-2305) and goat anti-rabbit HRP-conjugated secondary antibody (ZB-2301) were purchased from ZSGB-BIO (Beijing, China). The proteins were visualized using the Immobilon Western Chemiluminescent HRP Substrate Kit.

### Wound-healing assay

Cell migration was examined using the wound-healing assay. Briefly, the cells were plated and cultured to approximately 80-90% confluence in 6-well plates. A wound was created by scratching the cells vertically with a micropipette tip. The cells were washed 3 times with PBS to remove floating cells, and serum-free culture medium was added. A computer-based microscopy imaging system was used to determine wound healing at 0 h, 24 h and 48 h. The wound area was measured using ImageJ software in arbitrary units. The calculated methods of wound-healing assay were as follows: The mean wound width = area of wound/length of the wound, cell migration rate = (width of wound at 0 h-width of wound after incubation)/width of wound at 0 h *100%. A lower migration rate indicates a slower migration speed.

### Transwell assay

Briefly, 500 μL of DMEM containing 20% FBS was added to each well of the 24-well plate, followed by Transwell chamber (Corning, USA; 8 μM). Next, a 200 μL cell suspension (1×10^5^ cells) was added to the chamber. After 24 h incubation, the chamber was removed, and the cells on the upper chamber membrane were removed using a cotton swab. The migrated cells on the underside of the membrane were stained in 0.1% crystal violet for 15 min and washed with PBS 3 times. The number of migrated cells was counted in five random fields under a light microscope. For the cell invasion assay, a layer of artificially reconstituted basement membrane material Matrigel was coated on the bottom of the upper surface of the cell membrane (the dilution ratio of Matrigel to serum-free medium was 1:6).

### Cell immunofluorescence

Cells were seeded in a 24-well plate containing slides. When the cells were 50% confluent, the cell slides were fixed in 4% paraformaldehyde. Next, the slides were permeabilized with 0.2% Triton X-100. The slides were then incubated with E-cadherin (1:100; 3195; Cell Signaling) or Vimentin (1:100; 5741; Cell Signaling) antibody at 4 °C overnight. After 24 h, the cells were incubated with Alexa Fluor® 594-conjugated goat anti-rabbit secondary antibody (1:50; ZF-0316; ZXGB-BIO, China) in the dark for 2 h. For F-actin staining, phalloidin (Alexa Fluor™ 568 Phalloidin; A12380; Thermo Fisher) (500 μL of 1% BSA + 0.1 μL of phalloidin) was used to stain the cells in the dark at 37 °C for 1 h. Next, the nuclei were stained with DAPI for 5 min and washed with PBST 3 times. The slides were then sealed with glycerin and stored in the dark at 4 °C. The slides were observed under a fluorescence microscope.

### Co-immunoprecipitation

Weak RIPA buffer was used to extract cell proteins, and the protein supernatant was placed into 3 EP tubes at a ratio of 1:2.5:2.5. The first tube was used as input, and the IgG antibody (Normal Rabbit IgG, B900610, Proteintech or Normal Mouse IgG, SC2025, Santa Cruz) and target protein antibody were added to the last 2 tubes, followed by incubation with rotation overnight at 4 °C. The next day, magnetic beads were added and mixed for 2-3 h, and the expression of the corresponding protein was detected by Western blotting.

### Cell viability assay

The cell viability assay was assessed using the Cell Counting Kit 8 (CCK-8) according to the manufacturer's protocol. Briefly, the cells were seeded into a 96-well plate with 1,000 HeLa cells/well or 2,000 SiHa cells/well. Ten microliters of CCK-8 reagent was added the cells on the 1^st^, 2^nd^, 3^rd^, 4^th^ and 5th days. After 2 h incubation, the absorbance at 450 nm was measured.

### Tumor xenograft mouse model

Six-week-old female BALB/c athymic nude mice were subcutaneously injected into the left and right flanks with 3×10^6^ cells in 0.1 mL of PBS. Seven mice were included; the right flank of each mouse was injected with SiHa-sh-Rab31 cells, and the left flank was injected with control cells. Tumor growth was monitored, and the tumor volume was measured every 2 days with calipers. The tumor volume was calculated using the formula (L×W^2^)/2, where L is the length and W is the width of the tumor. Eighteen days after injection, the nude mice were euthanized and photographed using a small animal *in vivo* optical imager to observe the fluorescence intensity of subcutaneous tumors. Next, the tumors were isolated, fixed in formalin and embedded in paraffin. H&E staining and immunochemical staining of Rab31 and MAPK6 were performed. For the tumor metastatic assay, SiHa-sh-Rab31 cells or control cells were injected into the tail vein of nude mice, and lung and liver metastasis was determined on the 5th week. All the animal experimental procedures and protocols were approved by the Experimental Animal Ethics Committee of Shandong University School of Medicine.

### Statistical analysis

The data were expressed as means ± standard deviation (SD). Statistical analysis was performed using SPSS 20.0 (IBM, Chicago, IL, USA). The significant differences between the two groups were assessed by *two-tailed Student's t test*. Values of *P<*0.05 were considered statistically significant.

## Results

### Rab31 is upregulated in cervical cancer tissues and cell lines

**Figure 1:**
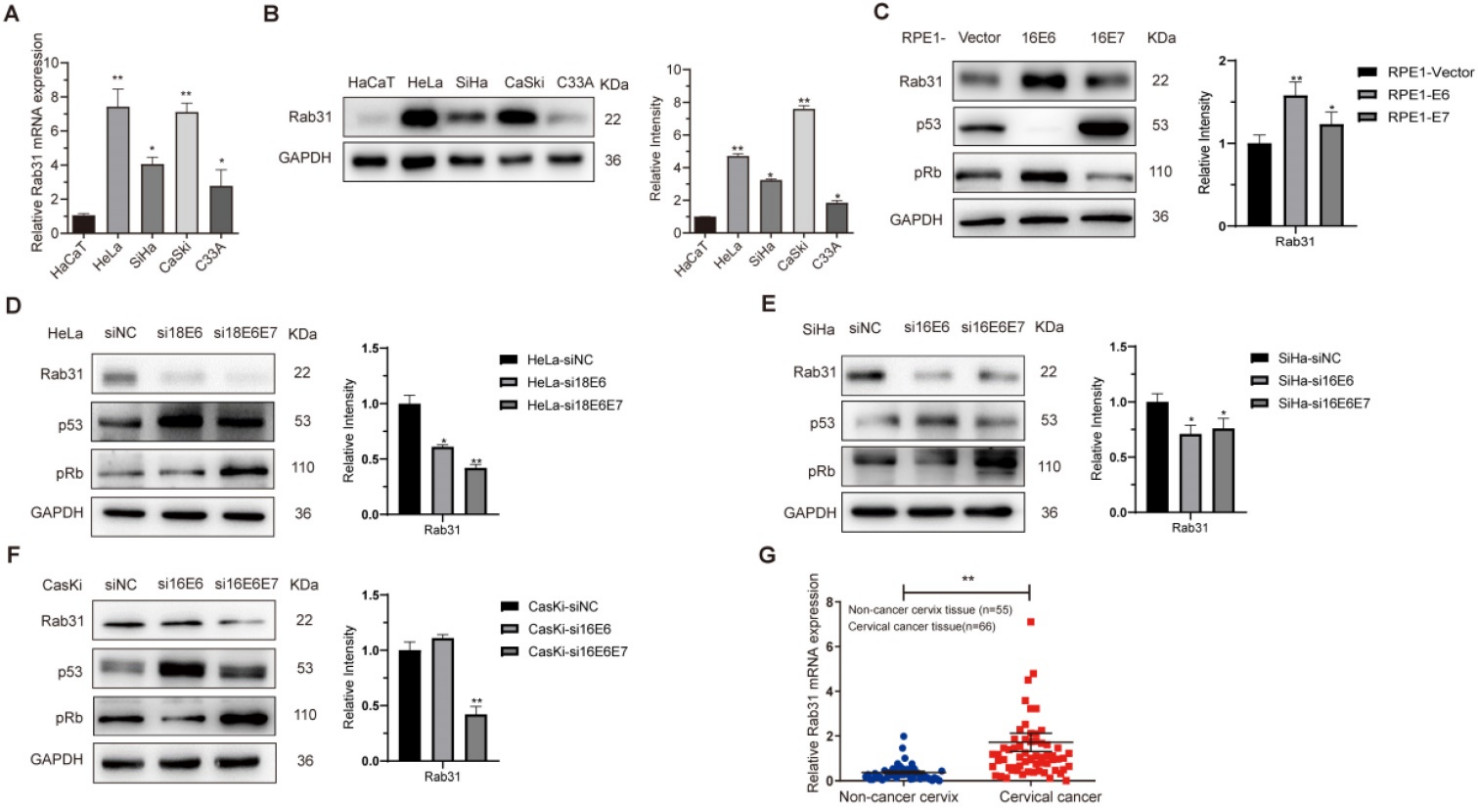
**Rab31 is highly expressed in cervical cancer cells and tissues. (A)** qRT-PCR was used to detect the mRNA level of Rab31 in different cells, and the mRNA level of Rab31 was higher in cervical cancer cells than in non-cancer HaCaT cells. **(B)** Rab31 protein level was detected by Western blotting, and Rab31 was highly expressed in cervical cancer cells. **(C)** Western blotting of Rab31, p53 and pRb in HPV16 E6- and E7-expressing RPE1 cells was shown. **(D-F)** Detection of Rab31, p53 and pRb protein levels in HeLa, SiHa and CaSki cells after transfection with siRNA targeting HPV18 E6, 18 E6E7 or 16 E6, 16 E6E7. **(G)** Detection of Rab31 mRNA level in 55 non-cancer cervical tissues and 66 cervical cancer tissue specimens using qRT-PCR, and Rab31 was highly expressed in cervical cancer tissues. *P <0.05, **P <0.01.

To explore the role of Rab31 in cervical cancer, we first examined the mRNA and protein expression levels of Rab31 in HaCaT and four cervical cancer cell lines, HeLa, SiHa, CaSki and C33A, using qRT-PCR and Western blotting, respectively. Both the transcription and protein levels of Rab31 were the lowest in non-cancer HaCaT cells and increased in cervical cancer cells (Figure 1A-B). Additionally, Rab31 expression was higher in HPV-positive cells than in HPV-negative C33A cells. Because the most important role in cervical carcinogenesis is attributed to viral oncoproteins E6 and E7, we investigated the effect of E6 and E7 on Rab31 expression. HPV E6 and E7 significantly promoted Rab31 expression (Figure 1C). Additionally, siRNA targeting HPV18 E6/18 E6E7 or 16 E6/16 E6E7 significantly decreased Rab31 expression in HPV18-positive HeLa or HPV16-positive SiHa and CaSki cervical cancer cells (Figure 1D-F). Because HPV E6 and E7 proteins are unstable and not easily detected, we examined target proteins of E6 and E7, such as p53 and pRb to prove the functional expression or efficient knockdown of E6 and E7. The construction of HPV E6/E7-overexpressing RPE1 cells and efficient knockdown of E6/E7 in cervical cancer cells were also proven previously 22. We further investigated the mRNA level of Rab31 in 55 non-cancer cervical tissues and 66 cervical cancer tissues collected from the Department of Pathology, Qilu Hospital of Shandong University. The expression of Rab31 in cervical cancer tissues was significantly higher than that in non-cancer tissues (Figure 1G). Taken together, these results suggest that Rab31 is highly expressed in cervical cancer tissues and cells and that both high-risk HPV E6 and E7 upregulate Rab31 expression.

### Rab31 promotes the migration and invasion of cervical cancer cells

**Figure 2:**
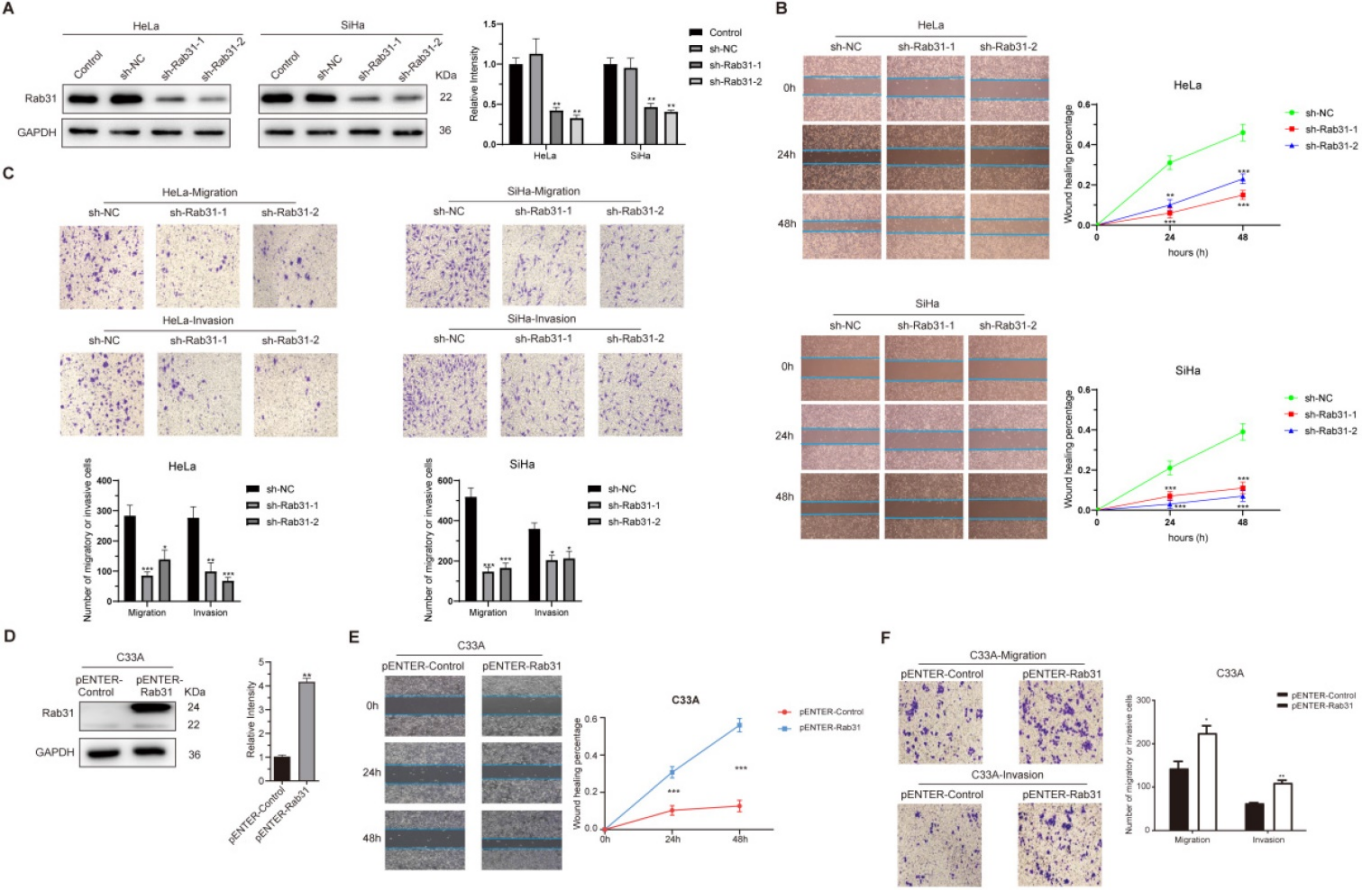
**Rab31 promotes the migration and invasion of cervical cancer cells. (A)** Two shRNA lentiviruses targeting Rab31 were used to infect HeLa and SiHa cervical cancer cells, and Western blotting showed that Rab31 was efficiently knocked down. **(B)** A wound-healing assay was used to detect the capability of cancer cells to migrate at 0 h, 24 h and 48 h. The statistical graph on the right is a quantitative analysis of the wound-healing measured at different time points. **(C)** The effect of Rab31 knockdown on cell migration and invasion was analyzed by Transwell assay. The pictures below showed the statistical results. **(D)** The Rab31 expression plasmid was transfected into C33A cells, and Rab31 expression was detected by Western blotting. **(E)** A wound-healing assay was performed and detected at 0 h, 24 h and 48 h. **(F)** The effect of Rab31 overexpression on cell migration and invasion was explored using Transwell assay. *P <0.05, **P <0.01, ***P <0.001.

Rab31 is highly expressed in HPV18-positive cervical cancer HeLa and HPV16-positive SiHa cells. To functionally analyze the role of Rab31 in cervical cancer cell lines *in vitro*, HeLa and SiHa cells were employed. Endogenous Rab31 was efficiently knocked down using lentiviral-based transfection (Figure 2A). Next, we explored the effect of Rab31 knockdown on the migration and invasion capabilities of cervical cancer cells. Rab31 knockdown notably inhibited the migration of HeLa and SiHa cells, and the wound was significantly increased compared with that of control cells at 24 h and 48 h (Figure 2B). Transwell assay showed that Rab31 knockdown inhibited the migration and invasion of cervical cancer cells (Figure 2C). Rab31 expression in HPV-negative cervical cancer C33A cells was relatively low. Therefore, we overexpressed Rab31 in C33A cells (Figure 2D). The results of wound-healing and Transwell assay showed that Rab31 overexpression greatly promoted the healing of the wound, migration and invasion abilities of C33A cells (Figure 2E-F). Taken together, these data indicate that Rab31 may act as an inducer of cell invasion in cervical cancer.

### Rab31 knockdown inhibits EMT and cytoskeletal rearrangement

**Figure 3:**
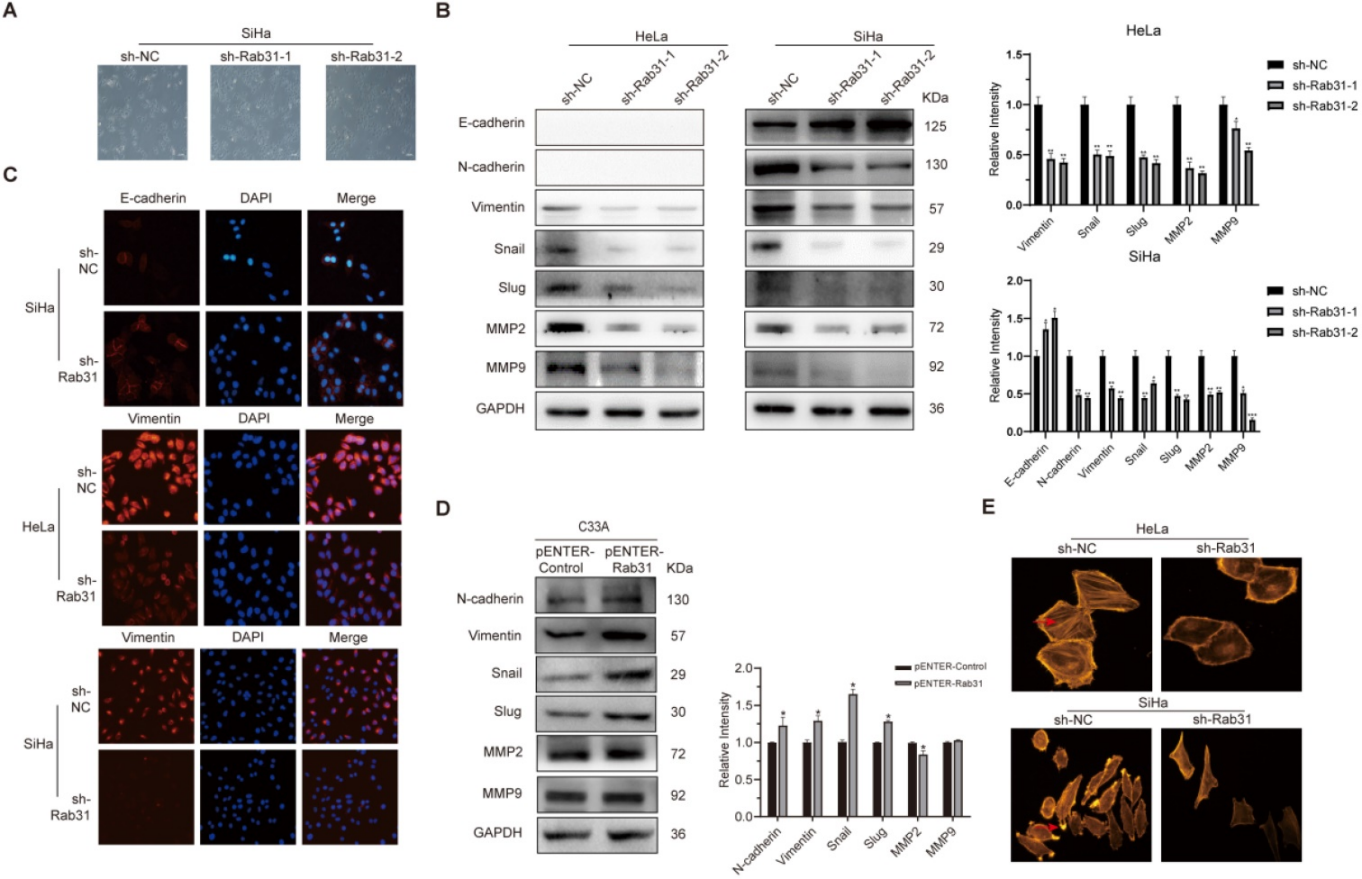
**Rab31 promotes the migration and invasion via EMT and cytoskeletal rearrangement. (A)** The effect of Rab31 knockdown on the morphology of SiHa cells was shown. **(B)** Western blotting of the protein levels of epithelial marker E-cadherin, mesenchymal markers (N-cadherin, Vimentin), EMT transcription factors Snail and Slug as well as MMP2, MMP9 in Rab31-knockdown HeLa and SiHa cells were shown. **(C)** Cellular immunofluorescence assays were performed to detect the expression of E-cadherin and Vimentin in HeLa and SiHa cells. **(D)** Expressions of EMT-related proteins in Rab31-overexpressing C33A cells were measured. **(E)** The filamentous actin backbone of HeLa and SiHa cells was fluorescently stained with phalloidin, and the cytoskeletal structure was observed under a fluorescence microscope. The arrow in the picture above indicates F-actin and the arrow in the picture below indicates the pseudopod. *P <0.05, **P <0.01, ***P <0.001.

EMT is closely associated with cancer cell metastasis capability, and EMT markers reflect the cell mesenchymal and epithelial status. Because Rab31 promoted the migration and invasion of cervical cancer cells, we detected the expression of classic EMT markers. Interestingly, Rab31 knockdown greatly changed the morphology of SiHa cells. The cells changed from spindle-shaped, fibroblast-like morphology to cobblestone-like epithelial-type morphology, but the morphology of HeLa cells did not change much (Figure 3A). Western blotting assays revealed that after Rab31 knockdown, the expression of the epithelial marker E-cadherin was increased, while that of the mesenchymal marker N-cadherin was decreased in SiHa cells (Figure 3B). However, no expression of E-cadherin and N-cadherin was detected in HeLa cells. The expression levels of Vimentin and the transcription factors Snail and Slug were reduced in both HeLa and SiHa cells. Matrix metalloproteinases MMP2 and MMP9 which degrade and remodel the extracellular matrix drive cell invasion and spread. The expressions levels of MMP2 and MMP9 were decreased following Rab31 knockdown. Consistent with the Western blotting data, immunofluorescence confirmed the high fluorescence intensity of E-cadherin in Rab31-knockdown SiHa cells (Figure 3C), while Vimentin expression in HeLa and SiHa cells was decreased. The expression levels of N-cadherin, Vimentin, Snail, Slug were all increased in Rab31-overexpressing C33A cells (Figure 3D). However, we did not detect the expression of E-cadherin in C33A cells, a finding that was consistent with previously published paper 23. The rearrangement of cell microfilament F-actin is one factor affecting cell migration. We showed that F-actin expression and the pseudopod were reduced with Rab31 knockdown (Figure 3E), indicating that Rab31 may regulate the rearrangement of the actin cytoskeleton to affect the capability of cell invasion.

### Rab31 interacts with MAPK6 and Rab31 inhibits MAPK6 degradation

**Figure 4:**
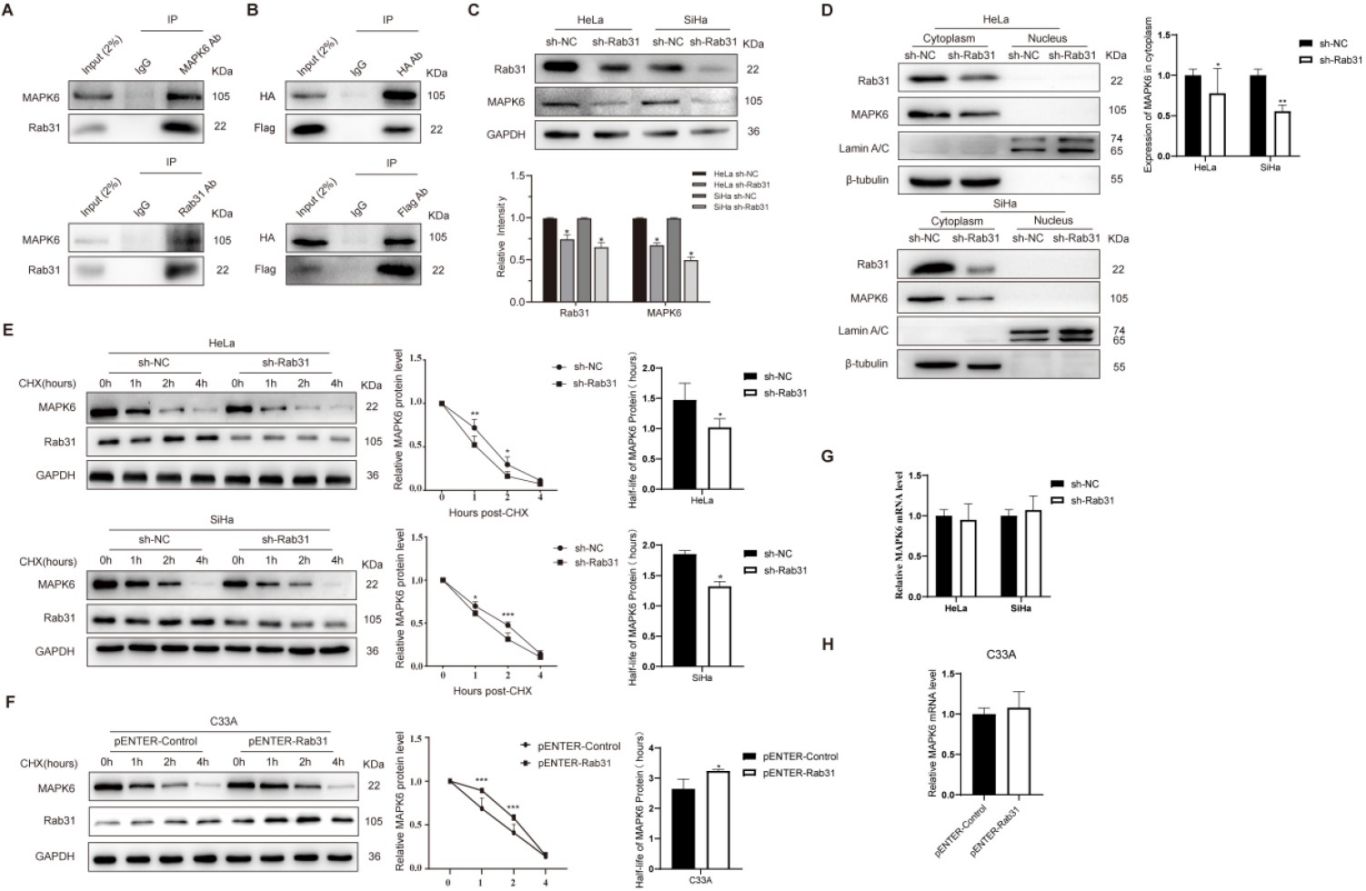
**Rab31 inhibits MAPK6 degradation. (A)** The interaction of endogenous Rab31 and MAPK6 in SiHa cells was detected using the Co-IP assay. **(B)** Transfection of plasmids expressing Rab31 (Flag tag) and MAPK6 (HA tag) into SiHa cells and the interaction between exogenous Rab31 and MAPK6 was detected. **(C)** Detection of MAPK6 protein expression after Rab31 knockdown. **(D)** Western blotting of MAPK6 cellular localization following Rab31 knockdown was shown. **(E)** Cells with Rab31 knockdown were incubated with 5 μg/mL of CHX and harvested at the indicated times. MAPK6 stability was monitored by the line chart, and the half-life of MAPK6 was evaluated on the right. **(F)** Cells with Rab31 overexpression were incubated with 5 μg/mL of CHX and harvested at the indicated times. MAPK6 stability and the half-life of MAPK6 were shown.** (G-H)** The mRNA level of MAPK6 was detected following Rab31 knockdown or Rab31 overexpression using qRT-PCR. *P <0.05, **P <0.01, ***P <0.001.

To investigate the mechanism by which Rab31 regulates the invasion of cervical cancer cells, we used the BioGRID database to search for molecules that interact with Rab31. One yeast two-hybrid assay showed that Rab31 interacted with MAPK6 in the BioGRID database. As expected, our co-immunoprecipitation (Co-IP) data showed an interaction between endogenous Rab31 and MAPK6 in SiHa cells (Figure 4A). To further validate the interaction between Rab31 and MAPK6, plasmids expressing Rab31 (Flag tag) and MAPK6 (HA tag) were transfected into SiHa cells and Co-IP was performed. Our results showed that exogenous Rab31 and MAPK6 bound to each other (Figure 4B). Additionally, MAPK6 expression was decreased when Rab31 was knocked down (Figure 4C). Next, a cell nucleoplasm fractionation assay was performed. MAPK6 protein was mainly localized in the cytoplasm and Rab31 knockdown resulted in a significant decrease in cytoplasmic MAPK6 in HeLa and SiHa cells (Figure 4D). One possible explanation is that Rab31 regulates MAPK6 degradation. To test this hypothesis, we detected MAPK6 protein stability after treatment with cycloheximide (CHX), which inhibits protein synthesis. The steady-state MAPK6 levels decreased more quickly with Rab31 knockdown in both HeLa and SiHa cells (Figure 4E), while the MAPK6 levels decreased more slowly with Rab31 overexpression in C33A cells (Figure 4F). The half-life of MAPK6 decreased significantly with Rab31 knockdown in HeLa and SiHa cells, while Rab31 overexpression in C33A cells markedly increased the half-life of MAPK6, indicating that Rab31 inhibits MAPK6 degradation. We also detected the mRNA level of MAPK6, and no significant change in the MAPK6 mRNA level could be observed upon Rab31 knockdown or overexpression (Figure 4G-H). These results suggest that Rab31 upregulates MAPK6 by increasing MAPK6 protein half-life and inhibiting its degradation.

### Rab31 promotes the migration and invasion of cervical cancer cells via MAPK6

**Figure 5:**
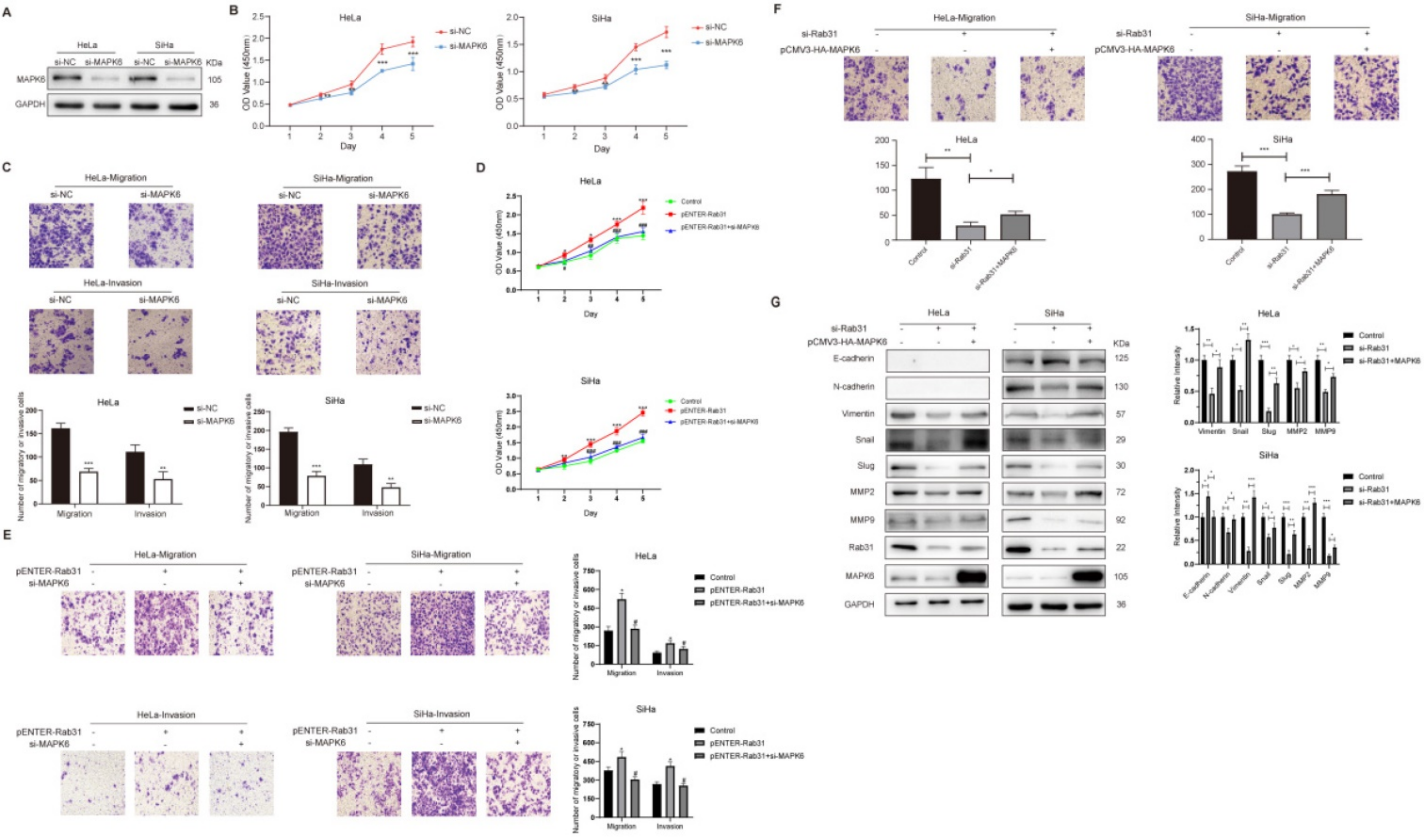
** Rab31 promotes the invasion of cervical cancer cells partly via MAPK6. (A)** Detection of MAPK6 protein in HeLa and SiHa cells after MAPK6 knockdown. **(B)** MAPK6 was knocked down in HeLa and SiHa cells, and CCK-8 assay was performed.** (C)** The migration and invasion of HeLa and SiHa cells were tested by Transwell assay following MAPK6 knockdown, and the pictures below show the statistical results.** (D)** The viability of Rab31-overexpressing cells following MAPK6 knockdown was analyzed by the CCK-8 assay. The significant difference of viability between Rab31-overexpressing cells with MAPK6 knockdown and Rab31-overexpressing cells was marked with *#.*** (E)** The migration and invasion of Rab31-overexpressing cells following MAPK6 knockdown were tested by Transwell assay. The pictures on the right show the statistical results. **(F)** The effect of MAPK6 overexpression on the metastatic capability of Rab31-knockdown HeLa and SiHa cells was detected using Transwell assay and the pictures below show the statistical results. **(G)** EMT-related proteins in Rab31-knockdown cells with MAPK6 overexpression were detected by Western blotting. *P <0.05, **P <0.01, ***P <0.001, #P <0.05,* ##P <*0.01,* ###P* <0.001.

To explore whether MAPK6 affected the viability, migration and invasion of cervical cancer cells, HeLa and SiHa cells were transfected with siRNA targeting MAPK6 (Figure 5A). The CCK-8 assay showed that cell viability was lower with MAPK6 knockdown (Figure 5B). Transwell assay showed that MAPK6 knockdown inhibited cervical cancer cell migration and invasion (Figure 5C). Additionally, MAPK6 knockdown eliminated the viability and migration/invasion effects of Rab31 overexpression (Figure 5D-E), suggesting that Rab31 is dependent on MAPK6 for these effects. We performed rescue experiments to further determine whether Rab31 promoted the invasion of cervical cancer cells in a MAPK6-dependent manner. We showed that MAPK6 overexpression partially restored the migration potential of Rab31-knockdown cervical cancer cells (Figure 5F). We also detected the expression of EMT-related proteins in these cells, and showed that MAPK6 overexpression inhibited E-cadherin expression, but partly rescued the expressions of mesenchymal markers N-cadherin, Vimentin, Snail and Slug, as well as MMP2 and MMP9 (Figure 5G). We conclude from these results that the pro-invasive effect of Rab31 in cervical cancer is partially dependent on MAPK6. In addition to MAPK6, other molecules or pathways may be responsible for Rab31-mediated migration and invasion of cervical cancer cells. The Hedgehog signaling pathway regulates the differentiation of human tissues/organs and is implicated in multiple cancers 24. Studies have shown that the Hedgehog signaling pathway is upregulated in osteosarcoma 25 and that Rab31 knockdown reduces the invasion capacity of osteosarcoma cells via inhibition of the Hedgehog signaling pathway 20. Hepatocyte growth factor (HGF), which is secreted predominantly by cancer-associated fibroblasts and present in the tumor microenvironment, promotes the survival and migration of cancer cells 26. Rab31 was reported to promote the migration of colon cancer cell lines by paracrine HGF, and HGF inhibition abolished the migration of cancer cells mediated by Rab31 expression 27.

### Rab31 knockdown inhibits tumor growth and metastasis in a xenograft nude mouse model

**Figure 6:**
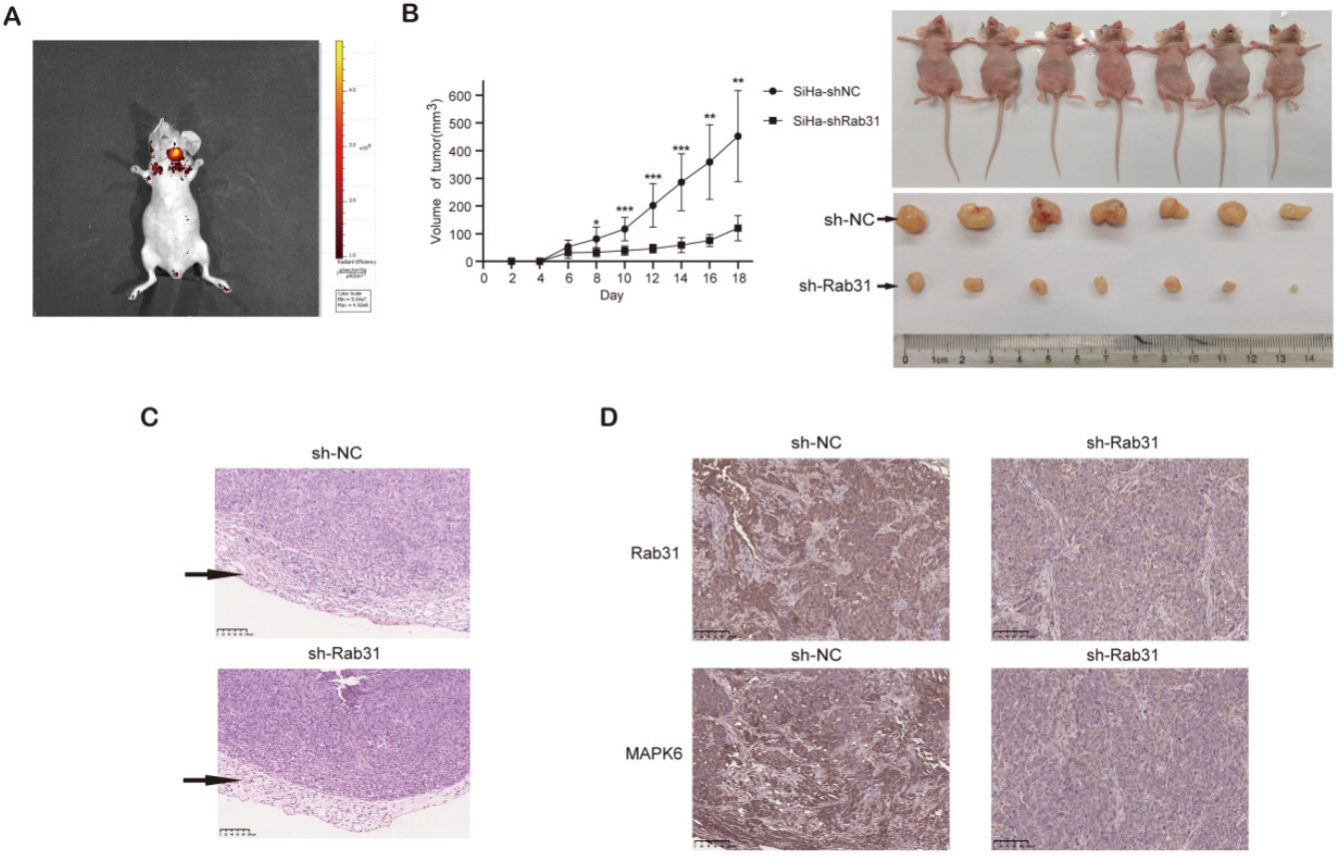
**Rab31 knockdown inhibits tumor growth in nude mice. (A)** *In vivo* fluorescence imaging of nude mice showed the fluorescence intensity of the tumors in the left and right flanks. **(B)** Tumor growth was monitored, and the volume was measured every 2 days with calipers. The growth curve of tumors was shown (left). The nude mice and tumors were photographed after the nude mice were euthanized (right). **(C)** H&E staining of transplanted tumor was used to detect the integrity of the tumor envelope. The arrows indicate the tumor envelope.** (D)** Immunohistochemical staining of Rab31 and MAPK6 in transplanted tumors was shown.^ *^P<0.05, ^**^P <0.01, ^***^P<0.001.

To further investigate whether Rab31 promotes tumor growth *in vivo*, Rab31-knockdown SiHa cells and control cells were injected into the right and left flanks of nude mice respectively. *In vivo* fluorescence imaging of nude mice showed that the fluorescence intensity of the tumor in the left flank was obviously stronger than that in the right flank (Figure 6A). Rab31 knockdown significantly inhibited the growth of tumors, as shown by the tumor growth curve (Figure 6B). Next, the mice were sacrificed and the tumors were separated, and Rab31 knockdown resulted in obviously smaller tumors than those in control mice. H&E staining of the transplanted tumors showed that the envelope was destroyed in the control group, while the envelope of the Rab31-knockdown group was intact (Figure 6C), indicating that Rab31 knockdown decrease the invasion of tumors. Immunohistochemical staining of transplanted tumors showed that the expression of Rab31 and MAPK6 in the Rab31-knockdown group was lower than that in the control group (Figure 6D). These results suggest that Rab31 promotes tumor growth *in vivo*.

**Figure 7:**
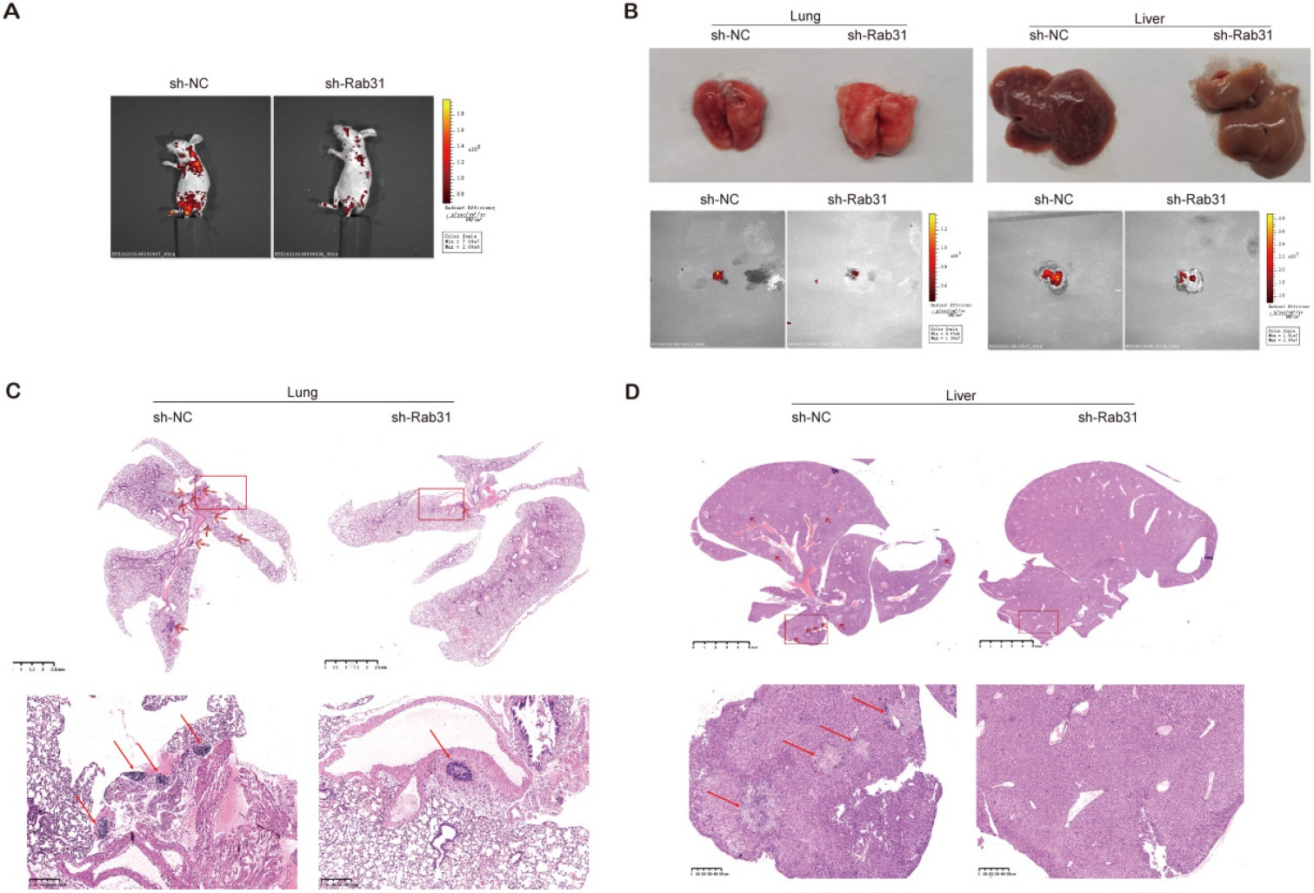
**Rab31 knockdown inhibits the metastasis of cervical cancer cells *in vivo*. (A)** *In vivo* fluorescence imaging of nude mice showed that fluorescence was mainly located in the chest and abdomen. **(B)** Visible lung and liver metastatic nodules and fluorescence imaging of the lung and liver were shown. **(C-D)** H&E staining of paraffin-embedded sections of lung and liver metastatic nodules was shown. The pictures below are enlargements of the rectangle in the pictures above. The arrows indicate the metastatic nodules.

To explore the effect of Rab31 on the metastatic capability of cervical cancer cells *in vivo*, we injected Rab31-knockdown SiHa cells and control cells into the tail vein to establish an *in vivo* tumor metastasis model. We showed that the fluorescence intensity of nude mice in the control group was obviously stronger than that in the Rab31-knockdown group, and the fluorescence was mainly distributed in the chest and abdomen (Figure 7A). Importantly, the incidence of lymph metastasis dramatically decreased in mice that were xenografted with Rab31-knockdown SiHa cells. Rab31 knockdown inhibited cervical cancer metastasis to the lungs and livers (Figure 7B). H&E staining was performed to further confirm the presence of lung and liver metastases (Figure 7C-D), and that lung and liver metastatic nodules were obviously decreased in Rab31-knockdown group. Overall, these data suggest that Rab31 is a key molecule for cervical cancer metastasis, and that Rab31 knockdown reduces the capability of cervical cancer cells to metastasize far *in vivo*.

## Discussion

Despite significant achievements in research on preventive HPV vaccines in recent years, cervical cancer remains a major public health problem. Many patients have invasion and metastasis of primary tumors, leading to a poor prognosis and even death. Therefore, exploring the molecular mechanism of cervical cancer invasion and metastasis is crucial.

In the present study, we revealed increased Rab31 expression in cervical cancer tissues and cells. Rab31 is highly expressed in breast cancer 16, gastric cancer 21, pancreatic cancer 19 and other malignancies. Additionally, Rab31 promotes the proliferation and metastasis of osteosarcoma cells 20. We showed that Rab31 level was higher in HPV-positive cervical cancer cells than in HPV-negative cervical cancer cells. Therefore, we used HPV-positive HeLa and SiHa cells as well as HPV-negative C33A cells to investigate the effect of Rab31 on cervical cancer invasion. We showed that Rab31 knockdown in HeLa and SiHa cells inhibited while Rab31 overexpression in C33A cells promoted the invasion of cervical cancer cells.

EMT enables epithelial cells to acquire a mesenchymal phenotype and enhances the capability of tumor cells to invade adjacent tissues and metastasize to distant organs, activities that play roles in tumorigenesis and progression 28. Epithelial markers include E-cadherin and tight junction proteins such as claudins. The loss of E-cadherin is regarded as a hallmark of cancer. Mesenchymal markers include N-cadherin, Vimentin and Snail 29. We showed that the expression of E-cadherin was increased when Rab31 was knocked down, while that of N-cadherin, Vimentin, Snail and Slug was decreased. Rab31 overexpression had the opposite effect on the expression of EMT-related proteins. These results revealed that cervical cancer cells activated EMT by upregulating Rab31. During metastasis, destruction of the extracellular matrix is a crucial step that requires the activation of proteolytic enzymes such as matrix metalloproteinases. Thus the overexpression of MMP2 and MMP9 also plays an essential role in EMT 30. Our results showed that the expression of MMP2 and MMP9 was reduced with Rab31 knockdown, decreasing the capability of cancer cells to metastasize. The cytoskeleton is a system of fibrillar structures that pervades the cytoplasm. The cytoskeleton comprises microtubules, microfilaments and intermediate filaments. It determines the morphology of cells, and cytoskeletal rearrangement is necessary for tumor metastasis 31. Pseudopods are important structures involved in the formation of actin cytoskeleton protrusions. They function as receptors of cells and communicate with the extracellular microenvironment. They also participate in cell adhesion and expansion 32. The results of F-actin staining showed that F-actin expression and the pseudopod were all decreased with Rab31 knockdown; thus, the capability of metastasis of cancer cells was reduced.

To explore how Rab31 promotes the invasion and metastasis of cervical cancer, we used the BioGRID database to search for the predicted molecules and found that Rab31 interacted with MAPK6 in a yeast two-hybrid system 33. MAPK6, also named extracellular signal-regulated kinase 3 (ERK3), has been proven to be upregulated in multiple cancers and to promote cancer cell migration/invasion. For example, MAPK6 is highly expressed in human lung carcinomas and promotes the invasion of lung cancer cells 34. Similarly, MAPK6 promotes the migration of head and neck cancer cells 35. We first verified the interaction between MAPK6 and Rab31. Next, we showed that Rab31 knockdown decreased the expression of MAPK6, which was localized in the cytoplasm. Further study revealed that Rab31 knockdown promoted while Rab31 overexpression inhibited the degradation of MAPK6. Whereas our study does not explore the underlying mechanism of MAPK6 degradation regulated by Rab31. We also investigated whether MAPK6 affected the development of cervical cancer and showed that MAPK6 knockdown inhibited the viability, migration and invasion of cervical cancer cells as well as Rab31-overexpressing cells. Additionally, the decrease in cell migration caused by Rab31 knockdown was rescued by MAPK6 overexpression, and the rescue expression of mesenchymal markers accounts for this effect.

The current study confirmed that Rab31 was aberrantly highly expressed in cervical cancer tissues and cells, and that both high-risk HPV E6 and E7 proteins upregulated Rab31. Rab31 promoted the invasion of cervical cancer cells by enhancing EMT and affected the cytoskeletal rearrangement. Additionally, Rab31 inhibited the degradation of MAPK6, and MAPK6 overexpression restored the reduced invasion capability of cervical cancer cells caused by Rab31 knockdown. Furthermore, Rab31 knockdown impaired tumor growth and metastasis via MAPK6 *in vivo*. Our study provides novel insights into the molecular mechanism of cervical cancer invasion and metastasis and suggests that Rab31 may be a new potential target in cervical cancer.

## Abbreviations

CCK-8: Cell Counting Kit 8
CHX: cycloheximide
DMEM: Dulbecco's modified Eagle medium
ECM: extracellular matrix
EGFR: epidermal growth factor receptor
EMT: epithelial-mesenchymal transition
ERK3: extracellular signal-regulated kinase 3
FBS: fetal bovine serum
GFP: green fluorescent protein
GLUT4: type 4 glucose transporter
H&E: hematoxylin and eosin
HGF: Hepatocyte growth factor
HPV: human papillomavirus
MAPK6: mitogen-activated protein kinase 6
MMP: matrix metalloproteinases
NC: negative control
NHERF1: Na^+^/H^+^ exchanger regulatory factor 1
PMSF: phenylmethanesulfonyl fluoride
qRT-PCR: quantitative RT-PCR
siRNA: small interference RNA
